# Increased Ingestion of Hydroxy-Methionine by Both Sows and Piglets Improves the Ability of the Progeny to Counteract LPS-Induced Hepatic and Splenic Injury with Potential Regulation of TLR4 and NOD Signaling

**DOI:** 10.3390/antiox11020321

**Published:** 2022-02-06

**Authors:** Meng Liu, Ying Zhang, Ke-Xin Cao, Ren-Gui Yang, Bao-Yang Xu, Wan-Po Zhang, Dolores I. Batonon-Alavo, Shu-Jun Zhang, Lv-Hui Sun

**Affiliations:** 1 Hubei Hongshan Laboratory, College of Animal Science and Technology, Huazhong Agricultural University, Wuhan 430070, China; liumeng0821@webmail.hzau.edu.cn (M.L.); zhangysunny@163.com (Y.Z.); caokexin131@hotmail.com (K.-X.C.); 2 Tang Ren Shen Group Co., Ltd., Zhuzhou 412007, China; yrg5068@trsgroup.cn; 3 College of Veterinary Medicine, Huazhong Agricultural University, Wuhan 430070, China; zwp@mail.hzau.edu.cn; 4 Adisseo France S.A.S., 10, Place du Général de Gaulle, 92160 Antony, France; dolores.batonon-alavo@adisseo.com; 5 Key Laboratory of Breeding and Reproduction of Ministry of Education, Huazhong Agricultural University, Wuhan 430070, China; sjxiaozhang@mail.hzau.edu.cn

**Keywords:** sows, piglets, methionine, lipopolysaccharide, tissue damage

## Abstract

Methionine, as an essential amino acid, play roles in antioxidant defense and the regulation of immune responses. This study was designed to determine the effects and mechanisms of increased consumption of methionine by sows and piglets on the capacity of the progeny to counteract lipopolysaccharide (LPS) challenge-induced injury in the liver and spleen of piglets. Primiparous sows (*n* = 10/diet) and their progeny were fed a diet that was adequate in sulfur amino acids (CON) or CON + 25% total sulfur amino acids as methionine from gestation day 85 to postnatal day 35. A total of ten male piglets were selected from each treatment and divided into 2 groups (*n* = 5/treatment) for a 2 × 2 factorial design [diets (CON, Methionine) and challenge (saline or LPS)] at 35 d old. After 24 h challenge, the piglets were euthanized to collect the liver and spleen for the histopathology, redox status, and gene expression analysis. The histopathological results showed that LPS challenge induced liver and spleen injury, while dietary methionine supplementation alleviated these damages that were induced by the LPS challenge. Furthermore, the LPS challenge also decreased the activities of GPX, SOD, and CAT and upregulated the mRNA and(or) protein expression of TLR4, MyD88, TRAF6, NOD1, NOD2, NF-kB, TNF-α, IL-8, p53, BCL2, and COX2 in the liver and (or) spleen. The alterations of GPX and SOD activities and the former nine genes were prevented or alleviated by the methionine supplementation. In conclusion, the maternal and neonatal dietary supplementation of methionine improved the ability of piglets to resist LPS challenge-induced liver and spleen injury, potentially through the increased antioxidant capacity and inhibition of TLR4 and NOD signaling pathway.

## 1. Introduction

Methionine is an essential amino acid for mammals and birds, and thus its dietary uptake is indispensable for animal maintenance, growth, and development [1]. It is the second or third most limiting amino acid for pigs that are fed corn-soybean meal diets [2,3]; it is used for protein synthesis and is involved in the methylation reactions of DNA and choline metabolism [4,5]. Additionally, methionine acts as the precursor for glutathione and taurine and plays a critical role in antioxidant defense and regulates immune responses in various animal species [6,7,8].

Weaning, when carried out at a very young age, is a stressful period psychologically and physiologically for piglets, which are susceptible to gastrointestinal diseases that are caused by pathogens, including pathogenic *Escherichia coli* (*E. coli*) [9]. The bacterial endotoxin lipopolysaccharide (LPS) can induce inflammation and oxidative stress, which causes damage to different organs and leads to adverse effects on animal productivity [10]. Our previous study showed that an increased consumption of methionine by both maternal and neonatal pigs can improve the capability of the progeny to cope with the LPS-induced negative effects [8]. However, the underlining mechanisms in this regard remain unclear.

Transmembrane toll-like receptors (TLRs) and cytoplasmic nucleotide-binding oligomerization domain proteins (NODs) play critical roles in modulating the innate and adaptive immune responses [11,12]. Toll-like receptor 4 (TLR4), the receptor of LPS, is a major player and triggers the activation of different intracellular signaling cascades, such as the activation of nuclear factor-κB (NF-κB) and the production of reactive oxygen species [13]. NOD1 and NOD2, as specialized NODs among the NOD family, which can connect with the peptidoglycan and LPS, and trigger the signal transduction pathway [14,15]. Therefore, TLRs or NODs can initiate a downstream signaling event that leads to the activation of NF-ĸB, which then stimulates the expression of inflammatory genes including interleukin (IL)-1β, IL-6, IL-8, and tumor necrosis factor-α (TNF-α). Consequently, the overproduction of proinflammatory cytokines can induce host tissue injury [13,14,15]. The main difference between TLRs and NODs is that TLRs are transmembrane sensors, and NODs are intracellular sensors [11]. Among the TLR family, TLR4 is the best studied member which responds primarily to LPS and induces an inflammatory response [12]. Among the NOD family, NOD1 and NOD2 are the best-characterized members, which can connect with the LPS and peptidoglycan, and trigger a signal transduction pathway [16].

Therefore, we hypothesize that increased the consumption of methionine by both sows and piglets might improve the capacity of the piglets to counteract LPS-induced injury to organs with the regulation of TLR4 and NOD signaling. The current study was conducted to investigate whether the increased consumption of methionine by both sows and progeny could alleviate the LPS-induced liver and spleen injury via the regulation of the TLR4 and NOD signaling in weaning pigs.

## 2. Materials and Methods

### 2.1. Animals, Treaztments, and Sample Collection

The animal protocol (HZAUSW-2018-022) of this study was approved by the Institutional Animal Care and Use Committee of Huazhong Agricultural University, China. In total, 20 primiparous sows (Landrace × Yorkshire) were divided into two groups (*n* = 10 sows/treatment) on day 85 of gestation based on their body weight and backfat thickness. A schematic of this experimental procedure to investigate the role of hydroxy-methionine (OH-Met) supplementation in piglets is shown in Supplemental Figure S1. Sows from the control group were fed a corn/soybean-control diet (CON), which was formulated to meet the nutritional requirements of sows (NRC, 2012, Table 1) [8,17]. Another group of sows were fed the control diet that was supplemented with OH-Met (Rhodimet AT88, Adisseo, France) at 25% above the total sulfur amino acids that were present in the CON. The detailed housing and feeding procedures for the sows were described in our previous study [8]. The feeding trial for the sows was lasted until the weaning of the piglets.

The piglets were weaned at the lactation day 21, and piglets from the same sow were kept in a pen. The piglets that were produced from the CON group of sows were fed a control diet which met the piglet’s nutrients recommendations (CON; NRC, 2012, Table 1) [8,17]. The piglets from the OH-Met group of sows were fed the CON that was supplemented with OH-Met at 25% above the total sulfur amino acids that were present in the CON [8]. The piglets were allowed free access to the feed and water. The feeding trial for the piglets was lasted 14 days. On the day 35, 20 male piglets from the two groups (10 piglets/group) were selected according to their average body weight. They were divided into 4 groups (*n* = 5 piglets/group) for a 2-by-2 factorial design trial that included the dietary treatments (CON and OH-Met) and immunological challenge [saline vs. LPS (100 μg/kg BW, *E. coli* 0111: B4, Sigma)] by intraperitoneal injection [8]. After 24 h post-challenge, the piglets were humanely euthanized by intravenous injection of sodium pentobarbital (40 mg/kg body weight) to collect the liver and spleen for histologic examination as previously described [18]. Meanwhile, the liver and spleen tissues were washed with ice-cold isotonic saline, snap-frozen in liquid nitrogen, and stored at −80 °C until use [19].

### 2.2. Histopathological and Redox Status Analysis

The liver and spleen tissues were examined microscopically after being fixed in 10% neutral buffered formalin, embedded in paraffin, sectioned at 5 µm, and stained with hematoxylin and eosin [18]. The total antioxidant capacity (T-AOC) and the activities of glutathione peroxidase (GPX), superoxide dismutase (SOD), and catalase (CAT), along with concentrations of malondialdehyde (MDA) were measured by specific assay kits (A003, A005, A001, A007–1, and A087–1–2) that were purchased from the Nanjing Jiancheng Bioengineering Institute of China. The protein concentrations were measured by the bicinchoninic acid assay.

### 2.3. Real-Time q-PCR and Western-Blot Analyses

Real-time q-PCR analyses of the pertaining samples were conducted as previously described [20]. The primer pairs that were designed using Primer Express 3.0 (Applied Biosystems) are shown in Table 2. The expression of the target genes relative to the housekeeping gene (β-actin) was analyzed by the 2^−ddCT^ method. The relative mRNA expression level of each target gene was normalized to the control group (as 1). Western blot analyses of the pertaining samples were performed as previously described [21]. The primary antibodies that were used for each gene product are presented in Supplemental Table S1. The protein concentrations were measured by the bicinchoninic acid assay.

### 2.4. Statistical Analysis

Statistical analysis was performed using SPSS (version 13, Chicago, IL, USA). The data are presented as the means ± SE. The data were analyzed by a two-way ANOVA with a significance level of *p* < 0.05. A Tukey test was used to do post hoc comparisons of the means if there was a significant effect.

## 3. Results

### 3.1. Liver and Spleen Histopathology

**Figure 1:**
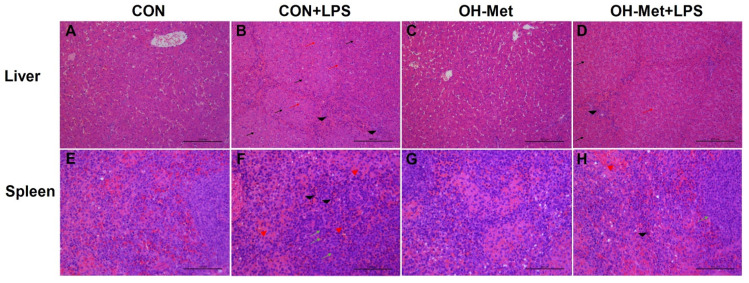
The effects of OH-Met supplementation on liver and spleen morphology after 24 h LPS-challenge in weaned piglets. The representative photomicrographs of liver and spleen sections that were stained with hematoxylin and eosin; photomicrographs are shown at 100× magnification (liver) and 200× magnification (spleen) magnification. Scale bars = 22.4 μm. The red arrow indicates narrowing of liver sinusoids; The black arrow indicates swelling; The black arrowhead indicates neutrophils infiltration; The red arrowhead indicates congestion; The green arrow indicates moderate lymphocytosis; CON, piglets receiving a control diet and injected with saline; CON+LPS, piglets receiving the control diet and challenged with LPS; OH-Met, piglets receiving a diet that was supplemented OH-Met at 25% above the total SAA present in the CON diet and injected with saline; OH-Met+LPS, piglets receiving a diet that was supplemented OH-Met at 25% above the total SAA present in the CON diet and challenged with LPS.

The histopathology of the liver and spleen are presented in Figure 1. Specifically, no obvious histopathological changes were observed in the liver and spleen of piglets in the control and OH-Met groups (Figure 1A,C,E,G). However, the histopathological results showed that LPS induced liver injury including cell swelling, narrowing of liver sinusoids, and an increase of neutrophil infiltration (Figure 1B). Meanwhile, LPS induced spleen damages such as congestion, moderate lymphocytosis, and neutrophilia infiltration in the splenic red pulp (Figure 1F). Notably, the dietary supplementation of OH-Met alleviated these LPS-induced damages in the liver (Figure 1D) and spleen (Figure 1H).

### 3.2. Antioxidant Parameters in Liver and Spleen

**Figure 2:**
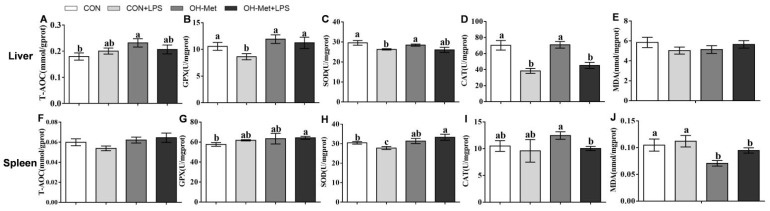
The effects of OH-Met supplementation on antioxidant indexes of the (**A**–**E**) liver and (**F**–**J**) spleen after 24 h LPS challenge in weaned piglets. The values are the means ± SEs, *n* = 5. Labeled means in a row without a common letter differ, *p* < 0.05. T-AOC, total antioxidant capacity; GPX, glutathione peroxidase; SOD, superoxide dismutase; CAT, catalase; MDA, malondialdehyde; CON, piglets receiving a control diet and injected with saline; CON+LPS, piglets receiving the control diet and challenged with LPS; OH-Met, piglets receiving a diet that was supplemented OH-Met at 25% above the total SAA present in the CON diet and injected with saline; OH-Met+LPS, piglets receiving a diet that was supplemented OH-Met at 25% above the total SAA present in the CON diet and challenged with LPS.

The antioxidant variables in the liver and spleen were significantly affected by the LPS challenge, dietary OH-Met supplementation, or their interaction (Figure 2). In the liver (Figure 2F–J), the LPS challenge did not affect (*p* ≥ 0.05) the activity of T-AOC and MDA concentration in both diets and decreased the activities of GPX and SOD only in the CON diet. It led to a 45.4% or 36.2% decrease (*p* < 0.05) in the CAT activity in both the diets with or without OH-Met. Notably, dietary OH-Met supplementation increased (*p* < 0.05) the hepatic GPX activity (30.5%) in the LPS-challenge groups. In the spleen (Figure 2F–J), LPS led to a 18.8% decrease in CAT activity (*p* < 0.05) in the dietary supplementation with OH-Met groups, while it only led to 8.8% decrease in the SOD activity (*p* < 0.05) in the diets without OH-Met supplementation. Notably, dietary OH-Met supplementation increased (*p* < 0.05) the splenic SOD activity (19.9%) in the LPS groups. The dietary OH-Met supplementation decreased (*p* < 0.05) the MDA concentration compared to the other three treatments that were injected or not with LPS.

### 3.3. Expression of TLR4 and NODs Signaling in Liver and Spleen

**Figure 3:**
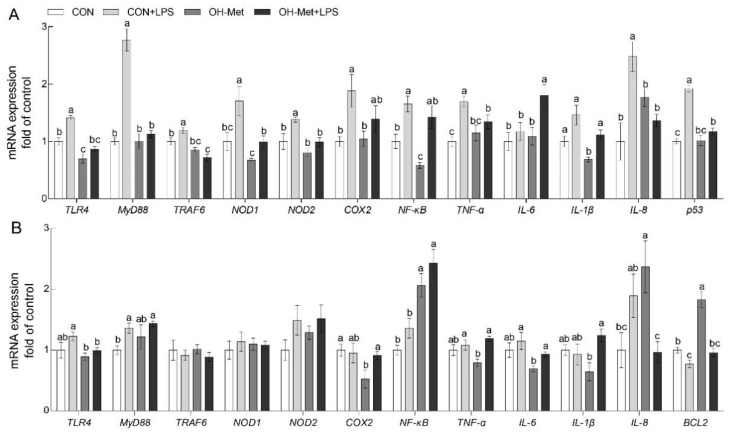
The effects of OH-Met supplementation on TLR4 and NODs signal-related genes of the (**A**) liver and (**B**) spleen after 24 h LPS challenge in weaned piglets. The values are the means ± SEs, *n* = 5. The labeled means without a common letter differ, *p* < 0.05. CON, piglets receiving a control diet and injected with saline; CON+LPS, piglets receiving the control diet and challenged with LPS; OH-Met, piglets receiving a diet that was supplemented OH-Met at 25% above the total SAA present in the CON diet and injected with saline; OH-Met+LPS, piglets receiving a diet that was supplemented OH-Met at 25% above the total SAA present in the CON diet and challenged with LPS.

Among the 12 assayed genes of TLR4 and NODs signaling, 12 genes in the liver and nine genes in the spleen were affected the LPS challenge, OH-Met supplementation, or their interaction (Figure 3). Specifically, the LPS challenge increased (*p* < 0.05) the mRNA levels of TLR4, myeloid differentiation factor 88 (*MyD88*), TNF-α receptor-associated factor 6 (TRAF6), NOD1, NOD2, NF-κB, cyclooxygenase 2 (COX2), TNF-α, IL-8, and tumor protein p53 (p53) in the liver of the CON groups (Figure 3A). Interestingly, the changes of 10 genes including TLR4, MyD88, TRAF6, NOD1, NOD2, NF-κB, COX2, TNF-α, IL-8, and p53 by the LPS challenge were prevented or mitigated (*p* < 0.05) by the OH-Met supplement (Figure 3A). In the spleen, LPS challenge increased (*p* < 0.05) the mRNA levels of MyD88 and decreased (*p* < 0.05) the mRNA levels of B-cell lymphoma 2 (BCL2) in the spleen of the CON and OH-Met groups (Figure 3B). Notably, dietary OH-Met supplementation alleviated (*p* < 0.05) the LPS-induced changes on the BCL2 expression in the spleen (Figure 3B). Meanwhile, the dietary OH-Met supplementation reduced (*p* < 0.05) TLR4 and IL-8 expression, as well as increased (*p* < 0.05) NF-κB expression (Figure 3B).

### 3.4. Production of Selected TLR4 and NODs Signaling Proteins in Liver and Spleen

**Figure 4:**
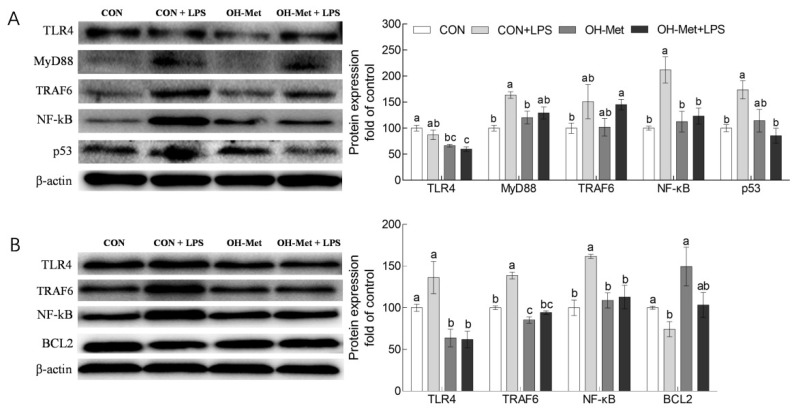
The effects of OH-Met supplementation onTLR4 and NODs signal-related protein production of the (**A**) liver and (**B**) spleen after 24 h LPS challenge in weaned piglets. The values are the means ± SEs, *n* = 3–4. The labeled means without a common letter differ, *p* < 0.05. CON, piglets receiving a control diet and injected with saline; CON+LPS, piglets receiving the control diet and challenged with LPS; OH-Met, piglets receiving a diet that was supplemented OH-Met at 25% above the total SAA present in the CON diet and injected with saline; OH-Met+LPS, piglets receiving a diet that was supplemented OH-Met at 25% above the total SAA present in the CON diet and challenged with LPS.

In the liver (Figure 4A), Western blot results showed that the LPS challenge increased (*p* < 0.05) the protein levels of MyD88, NF-κB, and p53 in the CON groups. Notably, the changes of MyD88, NF-κB, and p53 proteins by the LPS challenge were prevented or mitigated by OH-Met supplementation. Interestingly, the dietary OH-Met supplementation reduced (*p* < 0.05) the hepatic TLR4 protein in the LPS challenge groups. In the spleen (Figure 4B), Western blot results showed that LPS challenge increased (*p* < 0.05) the protein levels of TRAF6 and NF-κB but reduced (*p* < 0.05) the protein levels of BCL2 in the CON groups. Notably, the changes of these proteins by the LPS challenge were inhibited by OH-Met supplementation. Additionally, dietary OH-Met supplementation also reduced (*p* < 0.05) splenic TLR4 protein in both the saline and the LPS challenge groups.

## 4. Discussion

The interesting finding in this study was that the maternal and neonatal dietary methionine supplementation during the late gestation, lactation, and postweaning periods can mitigate the LPS challenge-induced damages in the liver and spleen of piglets. In this study, LPS challenge induced hepatic and splenic injury, as evidenced by swelling, sinusoidal narrowing, increased inflammatory cells, neutrophils in sinusoids and lobules of liver, as well as bruising, moderate lymphocytosis, and neutrophilia in the red pulp of spleen. These outcomes are consistent with previous studies, which provided evidence that damages in the liver and spleen were induced by LPS challenge [22,23,24]. Interestingly, the histopathological changes in the liver and spleen that were induced by the LPS challenge were moderated by dietary-supplemented OH-Met in this study. These findings are in line with our previous study which showed that methionine supplementation alleviated LPS challenge-induced negative changes on the plasma biochemistry biomarkers that were related to the liver function and inflammation of piglets [8]. Similar findings were also reported in cows and showed that methionine supplementation exerted protective effects against LPS challenge-induced negative effects in bovine mammary epithelial and polymorphonuclear cells [25,26].

Consistent with previous studies [27,28], the piglets that were challenged by LPS in the present study experienced oxidative stress, as indicated by the reduction of antioxidant capacity parameters (GPX, SOD, and T-AOC) and an increase of MDA concentration in the liver and/or spleen. Strikingly, the LPS challenge-induced imbalance in the redox status in the liver and the spleen were alleviated by the OH-Met supplementation in the current study. These outcomes were consistent with previous reports that methionine plays a particularly important role in providing Cys for GSH synthesis to improve antioxidant capacity [8,29,30]. Taken together, these results agreed with previous studies, which showed that dietary supplementation of methionine beyond sulfur amino acids growth requirements can reduce the LPS challenge-induced oxidative stress in pigs, poultry, and fish [8,31,32].

It is well documented that both the integral membrane TLRs and the cytosolic NODs are the receptors of LPS and play pivotal roles in the host defense against LPS-induced challenges [33,34]. In agreement with previous studies [33,34,35], the LPS challenge induced the mRNA and(or) protein production of TLRs (TLR4, MyD88, and TRAF6) and NODs (NOD1, NOD2) in the liver and (or) spleen of the piglets. Then, as the downstream signaling of TLRs and NODs, the inflammatory reaction-related genes including NF-kB, COX2, TNF-α, IL-6, IL-1β, and IL-8, were upregulated at the mRNA and (or) protein levels in the liver and (or) spleen of the piglets in the current study. Meanwhile, the LPS challenge also upregulated the apoptotic gene (p53) but downregulated anti-apoptotic gene (BCL2) at the mRNA and (or) protein levels in the liver and (or) spleen of the piglets. Consequently, the overproduction of pro-inflammatory cytokines and apoptosis-related signaling that was induced by the LPS challenge may be attributed to the damages in the liver and spleen of piglets that were observed in the present study [13,14,15,36]. Taken together, these outcomes are consistent with those that have been reported in previous studies [37,38], which showed that LPS challenge can induce tissue damage via the activation of TLRs and NODs signaling and further induce excessive inflammation and apoptosis. Strikingly, the increased consumption of sulfur amino acids, such as OH-Met, by sows and piglets prevented and (or) alleviated the changes of most of the genes in the liver and (or) spleen that were induced by the LPS challenge. These findings revealed that the increased consumption of methionine by sows and piglets alleviated the LPS challenge-induced damages in the liver and the spleen that were associated with the potential regulation of the TRLs and NODs signaling. In agreement with previous studies, methionine can downregulate TLR4 and (or) NODs signaling in osteoclast precursors and thus decrease bone loss during osteoporosis [39] in bovine mammary epithelial cells and improve the immune and antioxidant status [40]. Similar findings were also obtained for other amino acids in previous studies which showed that dietary supplementation of aspartate, glycine, and glutamate attenuate the LPS challenge-induced negative effects via TLR4 and (or) NODs signaling in tissues of piglets [41,42,43,44,45].

Nevertheless, several seemingly conflicting or inconsistent scenarios were observed in the current study. For example, the protein production of TLR4 and TRAF6 in the liver and NF-κB in the spleen between the saline and LPS challenge in CON diet groups, did not correlate well with their mRNA abundance. This discrepancy may be explained by a complex feedback or post-transcriptional mechanism regulating protein synthesis [38].

In summary, this study has illustrated that the maternal and neonatal dietary supplementation of OH-Met at 25% above the total sulfur amino acid requirements exert beneficial effects in alleviating the LPS challenged-induced damages in the liver and spleen of piglets. Moreover, the protective mechanism of OH-Met against LPS challenge-induced adverse effects may be associated with (1) an enhancement of the animal’s antioxidant capacities or (2) an inhibition of TLR4 and NOD signaling pathway. This finding indicated that the recommendations from NRC (2012) for sulfur amino acids for sows during gestation and lactation might require an update. Attention should also be paid to the provision of sulfur amino acids for weaned piglets that were often challenged by oxidation and inflammation.

## Figures and Tables

**Table 1:** Ingredients and nutrients composition ^1^.

Ingredients (%)	Sows		Piglets
	Gestation	Lactation	Post-Weaning
Corn	61.77	65.74	17.60
Expanded corn	-	-	15.0
Wheat flour	-	-	10.0
Wheat bran	15	-	-
Soybean meal	14	28	-
Expanded soybeans	-	-	8.0
Fermented soybean meal	-	-	5.0
Corn gluten feed	2.0	-	-
Fish meal	-	-	4.0
Whey powder	-	-	12.0
Soybean oil	3.5	2.5	-
Sugar	-	-	8.0
Glucose	-	-	6.0
Emulsified fat powder	-	-	5.0
Plasma protein	-	-	5.0
CaCO_3_	1.00	0.60	0.50
CaHPO_4_	1.20	1.70	1.50
Salt	0.30	0.30	0.30
DL-Met	0.07	0.06	0.30
L-Lys	0.16	0.10	0.50
L-Thr	-	-	0.30
Vitamin premix ^2^	0.50	0.50	0.50
Mineral premix ^3^	0.50	0.50	0.50
Crude protein (%)	14.6	17.6	21.0
Digestible energy (MJ/kg)	13.7	14.2	14.2
Total Lys (%)	0.75	0.98	1.45
Total Met (%)	0.29	0.32	0.48
Total Met + Cys (%)	0.52	0.60	0.82
D Lys (%)	0.65	0.85	1.30
SID Met (%)	0.26	0.28	0.45
SID Met + Cys (%)	0.45	0.52	0.72
Calcium (%)	0.69	0.69	0.65
Total phosphorus (%)	0.60	0.63	0.64

^1^ The OH-Met treatment diets during gestation, lactation, and days 21–35 were prepared by adding 1.477, 1.705, or 2.330 kg OH-Met (88%), respectively, to 1000 kg of the control diet at the expense of corn, to obtain TSSA levels in OH-Met treatments for gestation, lactation, and day 21–35 are 125% of the CON treatments, respectively. ^2^ Vitamin premix provided per kg of diet: retinyl acetate, 10,000 IU; dl-α-tocopheryl acetate, 50 IU; cholecalciferol 2500 IU; menadione, 5.0 mg; thiamin, 2.0 mg; pantothenic acid, 12.0 mg; riboflavin, 5.0 mg; pyridoxine, 10.0 mg; niacin, 30.0 mg; d-biotin, 0.2 mg; cyanocobalamin, 0.05 mg; folic acid, 1.5 mg; choline chloride 1500 mg. ^3^ Mineral premix provided per kg of diet: FeSO_4_·7H_2_O, 498 mg; ZnSO_4_·7H_2_O, 440 mg; CuSO_4_·5H_2_O, 78.7 mg; Na_2_SeO_3_, 0.66mg; MnSO_4_·5H_2_O, 110 mg; KI, 0.4 mg.

**Table 2:** List of primers that were used for q-PCR analysis ^1^.

Gene	Accession	Forward (5′-3′)	Reverse (5′-3′)
TLR4	NM_001113039.2	TGCTTTCTCCGGGTCACTTC	TTAGGAACCACCTGCACGC
MyD88	NM_001099923.1	GGCCCAGCATTGAAGAGGA	GACATCCAAGGGATGCTGCTA
TRAF6	NM_001105286.1	TTGGCTGCCATGAAAAGATGC	CTGAGCAACAGCCAGAGGAA
NOD1	NM_001114277.1	CAACCAAATCGGCGACGAAG	GCCGTTGAATGCAAGACTCAG
NOD2	NM_001105295.1	CTGTGAGCAGCTGCAGAAGT	TGGTTGTTTCCCAGCCTCAAT
NF-κB	NM_001048232.1	AGTACCCTGAGGCTATAACTCGC	TCCGCAATGGAGGAGAAGTC
COX2	NC_000845.1	ATGATCTACCCGCCTCACAC	AAAAGCAGCTCTGGGTCAAA
TNF-α	NM_214022.1	GGCCCAAGGACTCAGATCAT	CTGTCCCTCGGCTTTGACAT
IL-6	NM_214399.1	CCCTGAGGCAAAAGGGAAAGAA	CTCAGGTGCCCCAGCTACAT
IL-1β	NM_214055.1	CCCAATTCAGGGACCCTACC	TTTTGGGTGCAGCACTTCAT
IL-8	NM_213867.1	CTTCCAAACTGGCTGTTGCC	GTTGTTGTTGCTTCTCAGTTCTCT
p53	NM_213824.3	TGTAACCTGCACGTACTCCC	TCGGCCCGTAAATTCCCTTC
BCL2	XM_021099593.1	AGGATAACGGAGGCTGGGATG	CACTTATGGCCCAGATAGGCA
β-actin	XM_003124280.5	CTACACCGCTACCAGTTCGC	AGGGTCAGGATGCCTCTCTT

^1^ TLR4, toll-like receptor 4; MyD88, myeloid differentiation factor 88; TRAF6, TNF-α receptor-associated factor 6; NOD, nucleotide binding oligomerization domain containing; NF-κB, nuclear transcription factor kappaB; COX2, cyclooxygenase 2; TNF-α, tumor necrosis factor-α; IL, interleukin; p53, tumor protein p53; BCL2, B-cell lymphoma 2.

## Data Availability

Data are contained within the article.

## Supplementary Materials

The following are available online at https://www.mdpi.com/article/10.3390/antiox11020321/s1. Table S1: Name, type, dilution, and source of the primary antibodies; Figure S1: Schematic of the experimental procedure of the animal trial; Figure S2: Validation of the specificity of antibodies against and the correct bands of TRL4, MyD88, TRAF6, NF-κB, p53, and BCL2.
