# Supplementary material for: Increased Ingestion of Hydroxy-Methionine by Both Sows and Piglets Improves the Ability of the Progeny to Counteract LPS-Induced Hepatic and Splenic Injury with Potential Regulation of TLR4 and NOD Signaling
Source: Antioxidants (Basel). 2022 Feb 6;11(2):321. doi: 10.3390/antiox11020321 (PMC8868084; doi:10.3390/antiox11020321)
Supplement: Supplementary file 1 [file antioxidants-11-00321-s001.zip › antioxidants-1566778-supplementary.pdf]

Supplemental Material

Table S1 Name, type, dilution, and source of primary antibodies[1]

Antibody	Isotype	Dilution	Source
Primary Antibody			
TLR4	Rabbit	1:1000	ABclonal (Wuhan, China)
MyD88	Rabbit	1:1000	ABclonal (Wuhan, China)
TRAF6	Rabbit	1:1000	ABclonal (Wuhan, China)
NF-κB	Rabbit	1:1000	ABclonal (Wuhan, China)
p53	Rabbit	1:1000	ABclonal (Wuhan, China)
BCL2	Rabbit	1:1000	ABclonal (Wuhan, China)
β-actin	Rabbit	1:10000	ABclonal (Wuhan, China)
Secondary Antibody			
HRP labelled Antibody	Goat anti rabbit	1:10000	ABclonal (Wuhan, China)

[1]TLR4, toll like receptor 4; MyD88, myeloid differentiation factor 88; TRAF6, TNF-α receptor-associated factor 6; NF-κB, nuclear transcription factor kappaB; p53, tumor protein p53; BCL2, B-cell lymphoma 2.

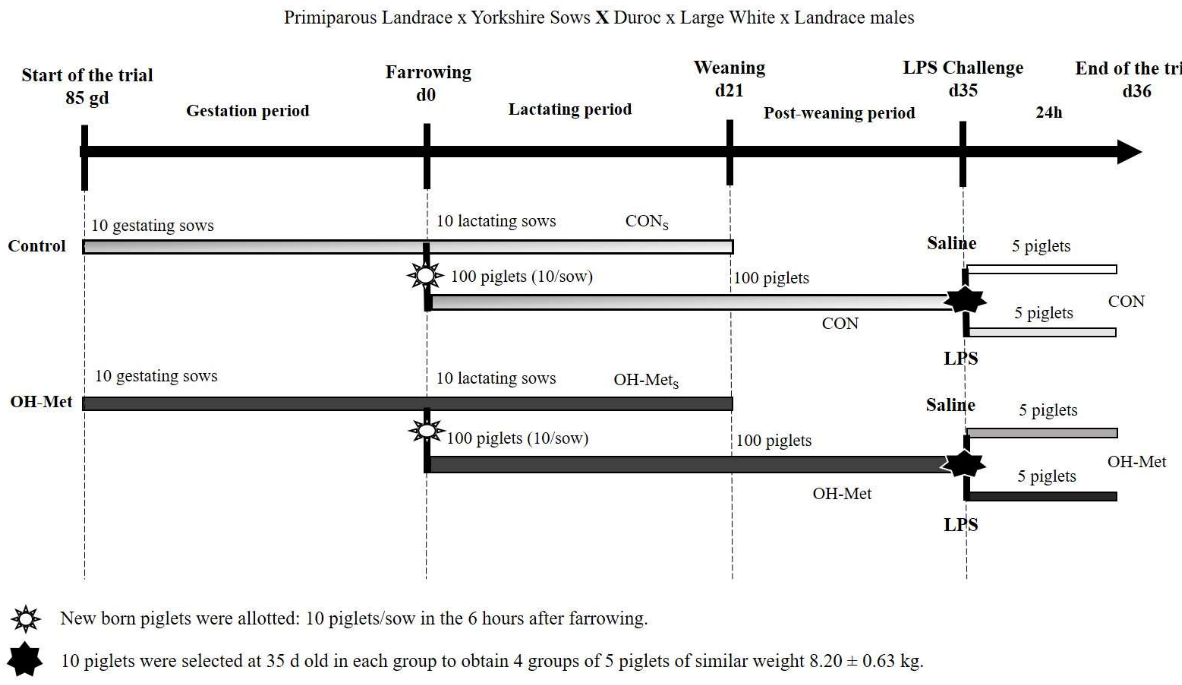

Figure S1 Schematic of the experimental procedure of the animal trial

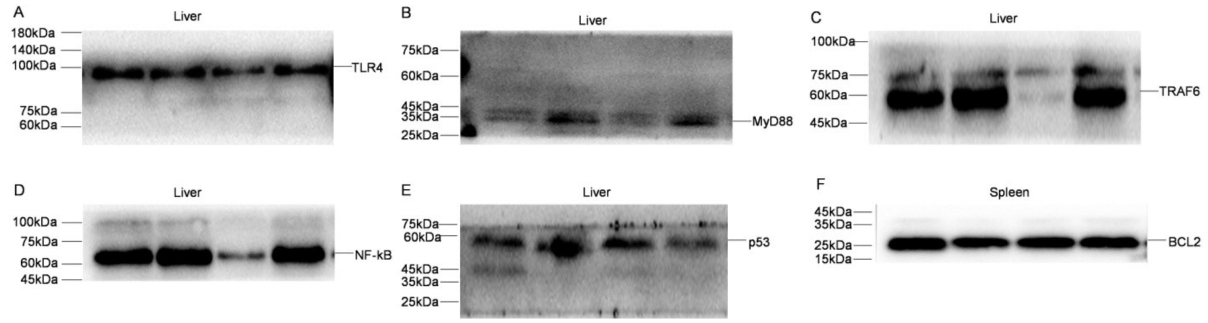

Figure S2 Validation of the specificity of antibodies against and the correct bands of TRL4, MyD88, TRAF6, NF-κB, p53, and BCL2. Western blot gel bands of TRL4 (A), MyD88 (B), TRAF6 (C), NF-κB (D) and p53 (E) were verified with piglet liver samples, and BCL2 (F) was verified with piglet spleen samples.
